# Glyceraldehyde-3-phosphate Dehydrogenase Common Peptides of *Listeria monocytogenes*, *Mycobacterium marinum* and *Streptococcus pneumoniae* as Universal Vaccines

**DOI:** 10.3390/vaccines9030269

**Published:** 2021-03-17

**Authors:** David Salcines-Cuevas, Hector Terán-Navarro, Ricardo Calderón-Gonzalez, Paula Torres-Rodriguez, Raquel Tobes, Manuel Fresno, Jorge Calvo-Montes, I. Concepción Pérez Del Molino-Bernal, Sonsoles Yañez-Diaz, Carmen Alvarez-Dominguez

**Affiliations:** 1 Grupo de Oncología y Nanovacunas, Instituto de Investigación Marqués de Valdecilla, 39011 Santander, Cantabria, Spain; davidsalcines@gmail.com (D.S.-C.); hteran35@hotmail.com (H.T.-N.); ricardocalderongonzalez@hotmail.com (R.C.-G.); paulatr98@hotmail.com (P.T.-R.); 2 Alamo Blanco, 18110 Granada, Andalucia, Spain; raquel.tobes@alamoblanco.org; 3 Centro de Biología Molecular Severo Ochoa, Universidad Autónoma de Madrid & DIOMUNE S.L., Parque Científico de Madrid, 28049 Madrid, Spain; mfresno@cbm.csic.es; 4 Servicio de Microbiología, Hospital Universitario Marqués de Valdecilla, 39008 Santander, Cantabria, Spain; jorge.calvo@scsalud.es (J.C.-M.); inmaculadaconcepc.perezdelmolino@scsalud.es (I.C.P.D.M.-B.); 5 Servicio de Dermatología, Hospital Universitario Marqués de Valdecilla, 39008 Santander, Cantabria, Spain; sonsolesjuana.yanez@scsalud.es; 6 Facultad de Educación y Facultad de Ciencias de la Salud, Universidad Internacional de La Rioja, Avda. La Paz 137, 26006 Logroño, La Rioja, Spain

**Keywords:** adjuvants, glyceraldehyde-3-phosphate-dehydrogenase, listeriosis, pneumonia, tuberculosis, vaccines

## Abstract

Universal vaccines can be prepared with antigens common to different pathogens. In this regard, the glyceraldehyde-3-phosphate dehydrogenase (GAPDH) is a common virulence factor among pathogenic bacteria of the genera Listeria, Mycobacterium and Streptococcus. Their N-terminal 22 amino acid peptides, GAPDH-L1 (*Listeria*), GAPDH-M1 (*Mycobacterium*) and GAPDH-S1 (*Streptococcus*), share 95–98.55% sequence homology, biochemical and MHC binding abilities and, therefore, are good candidates for universal vaccine designs. Here, we used dendritic cells (DC) as vaccine platforms to test GAPDH epitopes that conferred protection against *Listeria monocytogenes*, *Mycobacterium marinum* or *Streptococcus pneumoniae* in our search of epitopes for universal vaccines. DC loaded with GAPDH-L1, GAPDH-M1 or GAPDH-S1 peptides show high immunogenicity measured by the cellular DTH responses in mice, lacked toxicity and were capable of cross-protection immunity against mice infections with each one of the pathogens. Vaccine efficiency correlated with high titers of anti-GAPDH-L1 antibodies in sera of vaccinated mice, a Th1 cytokine pattern and high frequencies of GAPDH-L1-specific CD4+ and CD8+ T cells and IFN-γ producers in the spleens. We concluded that GAPDH-L1 peptide was the best epitope for universal vaccines in the *Listeria*, *Mycobacterium* or *Streptococcus* taxonomic groups, whose pathogenic strains caused relevant morbidities in adults and especially in the elderly.

## 1. Introduction

Dendritic cells (DC) are efficient experimental vaccine vectors as they can stimulate CD8^+^ and CD4^+^ T cells and induce Th1 cytokine profiles, relevant features of efficient vaccines for infectious diseases. In this regard, DC vaccines have been proposed against different viral and bacterial infections such as hepatitis C virus (HCV), human immunodeficiency virus (HIV), type 2 coronavirus (SARS-CoV2), *Streptococcus pneumonia, Chlamydia trachomatis*, *Mycobacterium tuberculosis* and *Listeria monocytogenes* [1,2,3,4,5,6,7,8,9,10].

Bacterial pathogens of the taxonomic groups *Listeria*, *Mycobacterium* or *Streptococcus* caused severe infections in adults and the elderly such as meningitis but also opportunistic skin diseases of varied severities [11,12,13,14,15,16,17,18]. European and WHO institutions are encouraging the science community to make efforts to prepare vaccines for adults. For this reason, we consider that vaccines able to offer protection against several bacteria that affect adults are interesting tools to explore as universal vaccines. 

Our hypothesis here follows previous studies of the group to search for multivalent or cross-reactive vaccines against the bacterial genera *Mycobacterium*, *Listeria* and *Streptococcus* spp. [19,20,21,22]. These studies are based on a common virulence factor, the glyceraldehyde-3-phosphate-dehydrogenase (GAPDH). GAPDH is a glycolytic enzyme that in Gram-positive bacteria (i.e., Listeria or Streptococcus) and mycobacteria presents analogous ADP-ribosylating abilities and MHC binding properties, that localized at the N-terminal 22 amino acid peptides (GAPDH_1-22_) with 98.5% sequence homology [19,23]. Here we explore the possibility that DC vaccines loaded with GAPDH_1–22_ peptides of Listeria monocytogenes (GAPDH-L1), Mycobacterium marinum (GAPDH-M1) or Streptococcus pneumoniae (GADPH-S1) acted as universal vaccines against these pathogens.

## 2. Materials and Methods

### 2.1. Bacteria, Peptide, Adjuvants and Cell Medium

We used *Listeria monocytogenes* strains 10403S (LM^WT^) as a control strain and a lysteriolysin O (LLO) deficient mutant of LM, LM-∆LLO derived from 10403S strain as non-pathogenic strain (gifts from D.A. Portnoy, Berkley University, CA, USA), *M. smegmatis* non-pathogenic mycobacteria strain (a gift from F.J Sangari and A. Seoane, IBBTEC-UC, Cantabria, Spain), S. pneumoniae non-pathogenic vaccine strain 49619-19F was obtained commercially from ATCC. *Listeria monocytogenes* (LM), *Mycobacterium marinum* (MM), *Mycobacterium chelonae* (MC), *Mycobacterium avium* (MA), *Mycobacterium tuberculosis* (MTB), *Streptococcus pneumoniae* (SP) (all of them serotype 5), *Streptococcus pyogenes* (SPY) and *Streptococcus agalactiae* (SA) were all clinical isolates of the Microbiology Department at our institution (Hospital Universitario Marqués de Valdecilla, Santander, Spain). Peptide from LLO virulence factor of *Listeria monocytogenes*, LLO_91–99_ is used as control peptide. Peptides from the GAPDH of *Listeria monocytogenes*: GAPDH_1–22_ (L1), GAPDH_23–45_ (L2) or GAPDH_315–337_ (Lc) peptides; from the GAPDH of *Mycobacterium marinum* GAPDH_8–29_ (M1) peptide or from the GAPDH of *Streptococcus pneumoniae* GAPDH_1–22_ (S1) peptide, were synthesized at Centro Nacional de Biotecnología (CSIC, Madrid, Spain) followed by HPLC and mass spectrometry using a MALDI-TOF Reflex IV spectrometer. Peptide purity was ≥99% after HPLC. Bone-marrow-derived dendritic cells (DCs) were obtained from femurs of 8–12-week-old female mice. BMDMs or DCs were cultured at 2 × 10^6^ cells/mL in six-well plates in Dulbecco’s Modified Eagle’s Medium (DMEM) supplemented with 20% foetal calf serum (FCS), 1 mM glutamine, 1 mM nonessential amino acids, 50 µg/mL gentamicin and 30 µg/mL vancomycin (DMEM complete medium) and 20 ng/mL granulocyte–macrophage colony-stimulating factor (GM-CSF) for DC, was added to the complete medium to obtain differentiated immune cells. On Day 7, the cells were harvested and analysed by fluorescence-activated cell sorting (FACS) to evaluate cell surface markers and appropriate differentiation of DCs using the following markers: CD11b–fluorescein isothiocyanate (FITC), CD11c–phycoerythrin (PE), IA^b^–allophycocyanin (APC), F4/80–PE, CD80–FITC, and CD86–V450 (BD Biosciences, Palo Alto, CA). Cells were collected using cell scrapers for detaching adherent cells. In certain samples, we also used after detachment, anti-mouse CD11c-coated magnetic beads and MACS^TM^ separation columns (Miltenyi Biotech, Auburn, CA, USA) on day 7 for positive selection. DC are cultured in DMEM complete medium with 10% FCS to a density of 10^10^ cells/mL and homogenized on Hepes-saline-EDTA buffer to obtain cytosol for ADP-ribosylation assays as reported [24,25]. DIO-1 is a TLR2/4 targeted molecule that we used as adjuvant [26,27].

### 2.2. Mice

We used C57BL/6 mice from our animal facilities at the University of Cantabria at 20–24 weeks old, an age that mimics in human beings 50 years of age and older. LD50 of *Listeria monocytogenes* strain 10403S in C57BL/6 mice is 2 × 10^5^ CFU/mice [7,20,28]. LD50 of LM (HUMV-01) was 2-fold higher 4 × 10^5^ CFU/mice. LD50 of *Mycobacterium marinum* (HUMV-MM01) is 2 × 10^4^ CFU/mice in C57BL/6 mice and LD50 of *Streptococcus pneumoniae* (HUMV-SP01) is 5 × 10^4^/mice in C57BL/6 mice. LD50 were evaluated in groups of mice (*n* = 10) i.v. infected with 2 × 10^4^ CFU/mice, 5 × 10^4^ CFU/mice or 10^5^ CFU/mice. Mice are examined for dead animals every 12 h and checked for clinical parameters of illness every 24 h.

### 2.3. Bioinformatics Analyses

GAPDH-LM, GAPDH-MM and GAPDH-SP BLASTP similarity searches were done via Internet using NCBI BLAST server (National Center for Biotechnology Information

8600 Rockville PikeBethesda MD, 20894 USA) (https://blast.ncbi.nlm.nih.gov/Blast.cgi?PROGRAM=blastp&PAGE_TYPE=BlastSearch) (accesed on 3 March 2021). The analysis of protein domains was based on Interpro (EMBL-EBI, Wellcome Genome Campus, Hinxton, Cambridgeshire, CB10 1SD, UK (https://www.ebi.ac.uk/interpro/) (accesed on 2 November 2020) [29,30]. Theoretical 3D predictive models were produced using the Automated Comparative Protein Modelling Server SWISSMODEL (Computational Structural Biology Group at the SIB Swiss Institute of Bioinformatics at the Biozentrum, University of Basel, Basel, Swizerland) (https://swissmodel.expasy.org/) (accesed on 20 December 2020) [31,32,33]. Peptide fold prediction was done using PEP-FOLD3 [34] (https://bioserv.rpbs.univ-paris-diderot.fr/services/PEP-FOLD3/) (accesed on 20 December 2020). The code used at PEP-FOLD3 server was:*python /service/env/PEPFOLD3.5/PEPFOLD3.py -s iSeq.data -l PEPFOLD --nRuns 100 --generator fbt --sortKey sOPEP --mcSteps 30000*. Multiple alignment and phylogenetic trees were done using CLUSTAL Omega at EBI server (https://www.ebi.ac.uk/Tools/msa/clustalo/) (accesed on 3 March 2021) [35]. Phylogenetic trees were obtained selecting Neighbour-joining algorithm without distance corrections. Explanations about the meaning of the colors in the alignment, the symbols used in the consensus line and about the phylogenetic trees are available at https://www.ebi.ac.uk/seqdb/confluence/display/JDSAT/Bioinformatics+Tools+FAQ (accesed on 3 March 2021). MHC predictions of peptide binding were performed with IEDB Consensus tool (www.iedb.org (accesed on 16 March 2021)) (Immune epitope database, National Institute of Allergy & Infectious Diseases, Bethesda, MD, USA) (accessed on 10 December 2020), indicating the binding epitopes to MHC class I and II molecules [36,37,38,39,40].

### 2.4. Recombinant Proteins Purification and Enzymatic and Quality Controls

*Escherichia coli* strain BL21 bearing plasmids to express large quantities of His-fusion recombinant full-length proteins of LLO (LLOrec) and GAPDH of *Listeria monocytogenes* (GAPDHrec) were obtained from Bioclone Inc (San Diego, CA, USA) or bearing a plasmid to express large quantities of GST-Rab5a. Expression of large quantities as His-fusion or GST-Rab5a proteins was induced with 1 mM IPTG for 5 h at 37 °C. His-tagged recombinant proteins were purified with TALON resin, according to the manufacturer’s instructions (Clontech, Takara Bio USA Inc., Mountain View, CA, USA) and GST-Rab5a protein was purified with glutathione beads (Clontech). To explore ADP-ribosylation abilities of GAPDH peptides onto GST-Rab5a, 3 µg of purified protein were incubated with 50 µM NAD-biotin at 37 °C in buffer 2X ADPRT (Tris-ClH 50 mM, pH 7.6, 10 mM ATP, 200 mM MgCl_2_, 20 mM NAD, 2 mM ADP-ribose) and 30 µg of a cytosolic extract of DC in the presence of the following sources: 3 µg of bacterial pathogen extracts (LM, MM or SP), *E. coli* (Ec) extract or different GAPDH-LM peptides of 22 amino acids, GAPDH_1–22_ (L1), GAPDH_23–45_ (L2) or GAPDH_315–337_ (Lc). Western blots are developed with Streptavidin-HRP [19,23,28].

Purification of recombinant proteins was evaluated after SDS-PAGE gels loading 3 µg of protein per lane and Coomasie stain as previously reported by our group [7,23]. Verification of protein purification was evaluated after cutting the bands from gels, TCA precipitation and proteomic identification at the Centro Nacional of Biotechnology (Madrid). Purified proteins were passed through the ToxinEraserTM kit (Genescript, Piscataway, NJ, USA) (catalog number L0038) to eliminate traces of endotoxin recombinant purified proteins and traces of endotoxin verified with the Genescript ToxiSensorTM chromogenic Limulus Amebocyte lysate kit (catalog number L0035C) (Genescript, Piscataway, NJ, USA). The endotoxin elimination kit consists of columns composed by an affinity matrix of modified polymyxin B. Endotoxin levels in protein purifications were lower than 0.1 EU/mL, according to the manufacturer. All proteins and peptides to be incubated with DC were tested for endotoxin traces and confirmed to have less than 0.1 EU/mL of endotoxin.

### 2.5. Preparation of DC Loaded with GAPDH Peptides and Assays of DC Activation

Bone-marrow cells (BM) were obtained from femurs of 24-week-old female mice. BM were cultured at 2 × 106 cells/mL in six-well plates in Dulbecco’s Modified Eagle Medium (DMEM) supplemented with 20% fetal calf serum (FCS), 1 mM glutamine, 1 mM non-essential amino acids, 50 µg/mL gentamicin and 30 µg/mL vancomycin (DMEM complete medium) and 20 ng/mL of granulocyte–macrophage colony-stimulating factor (GM-CSF) for seven days to differentiate BM to DC [1,2,6,7,20,28,29]. Differentiated DC have a phenotype of 98% CD11c^+^MHC-II^+^CD11b^−/+^CD40^−^86^−^ cells. These DC were used in vivo for delayed type hypersensitivity (DTH) assays (*see section below*), T cell responses or vaccination protocols. To activate DC, differentiated DCs are loaded with 50 μg/mL of recombinant proteins LLO_rec_, GAPDH_rec_ as positive controls or GAPDH peptides: GAPDH-L1, GAPDH-L2, GAPDH-Lc, GAPDH-M1 or GAPDH-S1 peptides, as well as with the listeriolysin O vaccine peptide, LLO_91–99_, as negative control [19] for 16 hours at 37 °C. Two adjuvants were included as controls of DC activation, LPS (10 ng/mL) or the Th1 adjuvant DIO-1 (10 ng/mL). Cell surface markers of DC were explored by flow cytometry to confirm or discard DC activation. Activated DC present the following phenotype: 90% of CD11c^+^IA^b+^CD40^+^CD86^+^ positive cells. Results are expressed as the percentage of positive cells ± SD of triplicate samples (*p* < 0.05). Activation was also examined after collection of DC supernatants after 16 hours incubation with peptides or other reagents, filtration and storage at −80°C to measure Th1-Th2 cytokine patterns using a multiparametric CBA kit of BD Biosciences (San Jose, CA, USA).

### 2.6. Immunoprecipitation of DC for Several GAPDH Domains

DC mice were infected with LM, MM or SP for 16 h. Cell lysates were immunoprecipitated with recombinant GST-Rab5a to explore proteins that bind to this small GTPase or with Blue-sepharose to explore proteins that bind to NAD domains. Immunoprecipitants were run onto SDS-PAGE and stained with Comassie to sequence eluted proteins or transferred to nitrocellulose membranes and incubated with primary antibody, rabbit anti-GAPDH-L1 and horseradish peroxidase-conjugated secondary antibodies. Immunoprecipitants were run onto SDS-PAGE, transferred to nitrocellulose membranes and incubated with primary antibody, rabbit anti-GAPDH-L1 and horseradish peroxidase-conjugated secondary antibodies.

### 2.7. Cell Toxicity and Apoptosis Assays of DC Vaccines

Activated DC were treated with or without different recombinant proteins or LLO and GAPDH peptides (50 µg/mL) for 16 h in culture medium and examined for cell toxicity or apoptosis. Cell toxicity is examined with Trypan-blue staining by light microscopy. Apoptosis is examined by FCAS using annexin-V conjugated to allophycocyanin (APC) fluorochrome and 7-AAD (7-aminoactinomycin D) (BD Biosciences, San Jose, CA, USA). Staining of cells with 7-AAD corresponded to necrotic cell death, whereas staining of cells with annexin-V alone corresponded to apoptotic programmed cell death (mean ± SD). Results are expressed as the % of cell toxicity or as the percentages of apoptotic cells ± SD of triplicate samples, respectively (*p* < 0.05).

### 2.8. DTH Responses Elicited by DC Loaded with Recombinant Proteins or Peptides

Female C57BL/6 mice were immunized intravenously (i.v.) with the following bacterial clinical isolates, LM (HUMV-LM01), MM (HUMV-MM01) or SP (HUMV-SP01) (5 × 10^3^ CFU). Seven days later, mice were injected with the different DC vaccines pre-loaded with recombinant proteins LLOrec or GAPDHrec or peptides, LLO_91–99_, LLO_294–304_, GAPDH-L1, GAPDH-L2, GAPD-Lc, GAPDH-M1 or GAPDH-S1 (10^6^ cells/mice) in the presence of DIO-1 (2 μg/mL) in left hind footpads, a procedure previously reported to explore the immunogenicity of vaccine epitopes [20]. Right hind footpads served as negative controls. Footpad thickness was measured with a caliper after 48 hours and results expressed in millimeters as the mean of three different experiments ± SD. To examine T cell responses in detail, popliteal lymph nodes of mice examined for DTH responses and cell homogenates were passed through a cell strainer to examine CD4^+^ and CD8^+^ T cell populations by flow cytometry. Results are expressed as the percentage of positive cells ± SD (*p* < 0.05).

### 2.9. Vaccination Assays with Activated DC Loaded with Different Peptides

Female C57BL/6 mice (*n* = 5/vaccine) were inoculated or not (*n* = 5) in the tail vain (i.v.) with one dose of 10^6^ DC pre-loaded with LLO_91–99_ (MHC-I restricted peptide of *L. monocytogenes*), GAPDH-L1 (GAPDH_1–22_ peptide of *Listeria monocytogenes*), GAPDH-M1 (GAPDH_8–29_ peptide of *Mycobacterium marinum*) or GAPDH-S1 (GAPDH_1–22_ peptide of *Streptococcus pneumoniae*), empty DC or saline. After 7 days, mice were challenged i.v. with LM (HUMV-LM01), MM (HUMV-MM01) or SP (HUMV-SP01) in saline (10^4^ CFU/mL). All animals were examined daily and 14 days later, mice were bled by ocular puncture and the blood was collected in ice-stored Eppendorf’s and kept on ice till processed to obtain sera. Sera are used to measure anti-GAPDH-L1 antibodies and cytokines. We did not include shorter times since humoral and cellular responses elicited by antigens, including antigens in vaccines, require at least 21 days total to be detected at significant levels. Mice are then killed to collect spleens to quantify viable bacteria in homogenized spleens (CFU/mL). Results are expressed as the percentages of protection ± SD of CFU/mL of vaccinated mice compared with mice inoculated with saline or empty DC that serve as controls after bacteria challenge. CFU of non-vaccinated mice are the following: saline LM (HUMV-LM01) 2.75 × 10^5^ CFU/mL, DC-CONT LM (HUMV-LM01) 2.60 × 10^5^ CFU/mL, saline MM (HUMV-MM01) 1 × 10^5^ CFU/mL, DC-CONT MM (HUMV-MM01) 0.9 × 10^5^ CFU/mL, saline SP (HUMV-SP01) 2.5 × 10^5^ CFU/mL, DC-CONT SP (HUMV-SP01) 2.49 × 10^5^ CFU/mL. Vaccination experiments were performed at least 10 times and we applied a *t* Student for statistics (*p* < 0.05).

### 2.10. Intracellular IFN-γ Staining 

Spleen cells of vaccinated and non-vaccinated mice were cultured in 96-well plates (5 × 10^6^ cells/mL) and stimulated for 5 h in the presence of brefeldin A and the following peptides: *Listeria monocytogenes* GAPDH-L1 peptide (vaccines DC-L1, DC-M1, DC-S1 and empty DC) (50 µM), LLO91-99 peptide (vaccines DC-LLO91-99), or for 5 hours in the presence of brefeldin A. Cells are surface labelled for CD4 or CD8, fixed and permeabilized with cytofix/cytoperm kit to measure IFN-γ (BD Biosciences). After sample acquisition by flow cytometry, data were gated for CD4^+^ or CD8^+^ events, and the percentages of these cells expressing IFN-γ are determined. Results are corrected according to the percentages of total CD4^+^ or CD8^+^ positive cells. Data were analyzed using FlowJo software (Treestar, Ashland, OR, USA). Results are expressed as the mean ± SD of triplicates (*p* < 0.05).

### 2.11. Frequencies of GAPDH-Specific CD8^+^ T Cells

We used recombinant soluble dimeric mouse H-2K^b^:Ig fusion proteins to determine the frequency of GAPDH-L1, GAPDH-S1 or GAPDH-S1-specific CD8^+^ T cells producing IFN-γ, following the manufacturer’s instructions (DimerX I, BD Bioscience) as described (7, 16). In brief, peptides (40 µM) were pre-incubated with H-2Kb:Ig (1 µM) in PBS for 16 h at 37 °C and next with the staining cocktail mix containing PE-conjugated A85-1 mouse monoclonal antibody for 1 hour at room temperature. Homogenized spleen cells (2 × 10^7^ cells/mL) were incubated with anti-IFN-γ and anti-CD8 antibodies together with the peptide mixed described above for 10 min at 4 °C. Percentages of CD8^+^ gated cells were expressed as the mean ± SD of triplicates (*p* < 0.05). Data were analyzed using FlowJo software.

### 2.12. Peptide ELISA Assay to Measure GAPDH-L1, GAPDH-M1 and GAPDH-S1 Titers

GAPDH-L1, GAPDH-M1, GAPDH-S1 peptides (50 μg/mL) were coated to 96-well plates in carbonate buffer (pH 8.0) overnight at 4 °C. Plates were washed and incubated with 1 mg/ml of BSA (fraction V) to saturate all sites in the plates. Sera of vaccinated or non-vaccinated mice were 1/10 diluted and peptide coated plates incubated with diluted sera for 2 h at RT as described (7, 22). Reactions were developed with goat anti-human IgG or goat anti-mouse IgG and absorbance analyzed at 450 nm. Results are presented as optical units (OD) and mean values ± SD of triplicate experiments (*p* < 0.05).

### 2.13. FACS Analysis

Cell surface markers of murine DC or murine spleens were analyzed by FACS using the following antibodies: anti-HLA-DR-FITC, anti-CD45-PerCP, anti-CD14-PE and anti-CD86-V450 (clone 2331). For cell surface markers of murine DC, we used biotin anti-IAb (clone AF6-120-1), anti-CD11c-PE (clone HL3), anti-CD40-APC (monoclonal 3/23 from BD Pharmingen (BD Biosciences, San Jose, CA, USA)), anti-CD86-V450 (clone GL-1) and for murine spleens we also used anti-CD4-FITC (clone RPA-T4) and anti-CD8-PE (clone RPA-T8) (BD Biosciences). All samples were treated with propidium iodide to gate dead cells. Flow cytometry was performed with a FACSCalibur (BD Biosciences, San Jose, CA, USA) and data were analyzed using the FlowJo software.

### 2.14. Cytokine Analysis

Cytokines in mice sera or DC supernatants were quantified using multiparametric CBA kits, either for mice or for human samples (BD Biosciences, San Jose, CA, USA). Mouse Th1/Th2/Th17 CBA kit (catalog number 560485) was used to measure cytokines in mice sera and DC supernatants. Cytokine concentrations are expressed as the average of three replicates in pg/mL ± SD. Data were analyzed using the FlowJo software.

### 2.15. Statistical Analysis

For statistical analysis, Student’s *t*-test was applied to all mice assays infected with bacterial pathogens since sample sizes were > 4. In fact, two groups of 5 mice per bacterial strain were inoculated and a *p*-value < 0.05 was considered significant. For flow cytometry analysis (cytokine, cell surface markers or intracellular IFN-γ staining) performed in triplicate, we employed the ANOVA test and a *p*-value < 0.05 was considered significant. GraphPad software was used for generation of all graphs presented.

### 2.16. Ethics Statement

This study was carried out in accordance with the Guide for the Care and Use of Laboratory Animals of the Spanish Ministry of Science, Research and Innovation. The Committee on the Ethics of Animal Experiments of the University of Cantabria approved the protocol (Permit Number: PI-01-17) that follows the Spanish legislation (RD 1201/2005). All surgeries were performed by cervical dislocation, and all efforts were made to minimize suffering. Similarly, for the use of human data of bacteria clinical isolate, we have a positive approved project from the Committee of Clinical Ethics of Cantabria (CEm) entitled: “Clinical Development of Listeria based vaccines” that includes Informed Consent and General Project Information documents to patients (Permit Acta Number: 29/2014, internal code: 2014.228).

## 3. Results and Discussion

We started this work with the hypothesis that DC vaccines loaded with the N-terminal peptides of GAPDH from the bacterial taxonomic groups of Listeria, Mycobacterium and Streptococcus, might serve as universal vaccines to explore epitopes that confer broad protection against pathogenic strains of these genera.

First, we performed a bioinformatic study at our health institution in adults elder than 50 years that presented pulmonary, meningitis or cutaneous infections from 2014 to 2018, and focused on GAPDH sequences. We observed that only clinical isolates of the taxonomic groups of Listeria, Mycobacterium and Streptococcus presented GAPDH sequences with homologies higher than 90% (Table 1, column 1). While streptococcal infections are the most prevalent ones in Cantabria with annual incidences of 77 cases per 100,000 inhabitants, the incidences of listeriosis and mycobacteria were lower but significant, with 4.4 and 1.41 cases per 100,000 inhabitants, respectively. These annual incidences, together with GAPDH homologies higher than 90%, justified preparing universal vaccines for adults based in a single antigen as GAPDH [23].

We noticed that clinical isolates of year 2016 included bacterial strains from all the taxonomic groups of Table 1 (column 2), and, therefore, we selected these bacterial strains for further studies and assigned them an internal code for privacy: HUMV-LM01 to HUMV-LM07 for *Listeria monocytogenes* (LM), HUMV-MM01 for *Mycobacterium marinum* (MM), HUMV-MTB01 for *Mycobacterium tuberculosis*, HUMV-MA01 for *Mycobacterium avium*, HUMV-MC01 to HUMV-MC05 for *Mycobacterium chelonae* (MC), HUMV-SP01 to HUMV-SP03 for *Streptococcus pneumoniae* (SP), HUMV-SA01 to HUMV-SA03 for *Streptococcus agalactiae* (SA) and HUMV-SPY01 to HUMV-SPY03 for *Streptococcus pyogenes* (SPY) (Supplementary Table S1). We next evaluated the virulence of these clinical isolates in mice and tried to identify immunological biomarkers related to GAPDH immune responses. Since streptococcal cases were more frequent than listeriosis or mycobacteria cases, for comparison purposes, we selected similar numbers of bacterial strains: 7 cases of listeriosis (LM), 8 cases of mycobacteria (MTB, MA, MM and MC) and 9 cases of Streptococcus (SA, SP and SPY). We inoculated intravenously (i.v.) two groups of 5 mice per bacterial strain (*n* = 5). Since humoral immune responses require 14 days to detect IgG levels in sera, we performed virulence assays for 14 days, using same bacterial inoculum per mice, 10^4^ CFU/mice, that corresponded to at least 2-fold LD50 of each strain. 

Our results (Supplementary Table S1) indicated that four clinical isolates, HUMV-LM01 of LM; HUMV-MTB01 of MTB, HUMV-MM01 of MM, and HUMV-SP01 of SP, can be considered hypervirulent strains since they met the criteria of at least 100-fold higher CFU/mL values in spleens than non-pathogenic strains, and CFU values that showed correlation with high anti-GAPDH immune responses (i.e., high titers in mice sera of anti-GAPDH-L1 antibodies with OD ≥ 2.0, as well as significant induction of CD4 and CD8 responses) (asterisks data in Supplementary Table S1). Non-pathogenic strains used as basal controls of each taxonomic group were, listeriolysin O deficient strain (LM-∆LLO) for LM clinical isolates, *Mycobacterium smegmatis* for all mycobacteria clinical isolates and *Streptococcus pneumoniae* vaccine strain 49619-19F for Streptococcus clinical isolates. We selected these hypervirulent strains for the vaccination studies, HUMV-LM01, HUMV-MM01 and HUMV-SP01, to evaluate the efficacy of the vaccine platforms in the more adverse conditions.

### 3.1. Selection of the Antigenic Epitope of Listeria Monocytogenes, Mycobacterium Marinum and Streptococcus Pneumoniae for the Vaccine Platforms

Once we have established the experimental infection models in mice, strains, route of inoculation, number of CFU/mice for the vaccination assays, we explored the best antigenic epitope. Since previous studies indicated that GAPDH from the taxonomic groups of *Listeria*, *Mycobacterium* and *Streptococcus* contained a NAD-binding domain at the N-terminal domain that also shared immunogenic domains [19,21,22], we selected the GAPDH N-terminal domain of these bacteria as a putative epitope to focus our vaccine designs. To confirm that the bacterial clinical isolates selected for the vaccination assays contained this GAPDH epitope at the N-terminal, we isolated GAPDH from DC infected with the bacterial clinical isolates, HUMV-LM01, HUMV-MM01 or HUMV-SP01 and performed biochemical and immunogenic assays. We confirmed that isolated GAPDH presented similar Rab5a- and NAD-binding domains (panels (a) and (b) in Supplementary Figure S1). We also validated that GAPDH, purified protein or in lysates of DC infected with these clinical isolates, was detected with the polyclonal rabbit anti-GAPDH-L1 antibody that recognizes amino acids 1–22 of the NAD-binding domain (panels (c) and (d) and text in Supplementary Figure S1). We concluded that N-terminal domain of GAPDH with a reported 30 amino acid length [23] contained common epitopes of *Listeria*, *Mycobacterium* and *Streptococcus* clinical isolates to include in vaccine designs.

### 3.2. Adjuvant and Immunogenic Abilities Elicited by DC Loaded with GAPHD-L1, GAPDH-M1 or GAPDH-S1 Peptides

**Figure 1:**
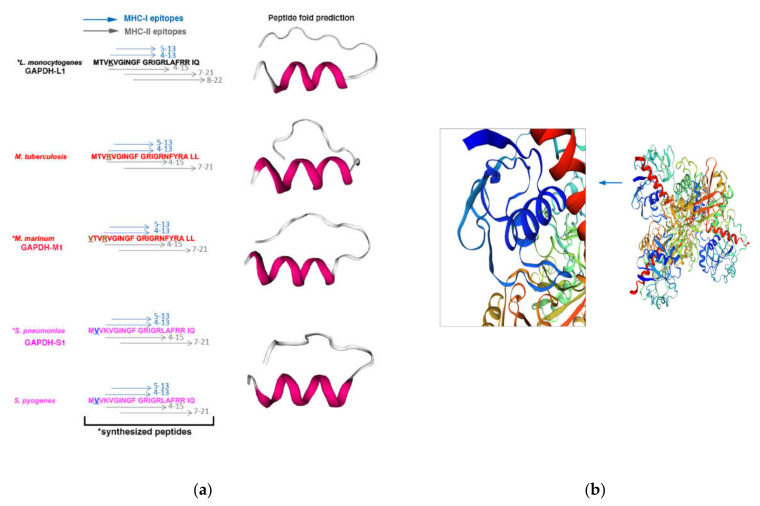
Predicted MHC-I and MHC-II binding epitopes of GAPDH peptides, peptide folding and cristal structures of *Listeria monocytogenes*, *Mycobacterium marinum* and *Streptococcus pneumoniae* and. (**a**) On the left, we present the predicted binding epitopes of the synthesized GAPDH peptides (asterisks) to MHC-I and MHC-II molecules, using the IEDB consensus tool. Blue arrows indicate the residues binding to MHC-I molecules and grey arrows, the residues binding to MHC-II molecules. On the right, we show GAPDH peptides fold predictions using the PEP-FOLD3 system. (**b**) Predicted crystal of GAPDH-LM homotetramer using the SWISSMODEL server. GAPDH-L1 peptide is shown in dark blue and in the enlarged image. Predicted 3D images of crystal structures are similar to GAPDH peptide folding predictions in panel (**a**).

Next, we designed peptides of 22 amino acids length of *Listeria monocytogenes* (LM) (GAPDH_1–22_ that we called GAPDH-L1), *Mycobacterium marinum* (MM) (GAPDH_8–29_ that we called GAPDH-M1) and *Streptococcus pneumoniae* (SP) (GAPDH_1–22_ that we called GAPDH-S1) and synthesized them at 99% purity (Centro Nacional de Biotecnologia, Madrid, Spain). Using bioinformatic approaches for peptide fold predictions as PEP-FOLD3 (python/service/env/PEPFOLD3.5/PEPFOLD3.py -s iSeq.data -I PEPFOLD –nRuns 100 –generator fbt –sortKey sOPEp –mcSteps 300000), we predicted that peptide folding of GAPDH-L1, GAPDH-M1, GAPDH-S1 provided similar structures (right images in Figure 1, panel (a) [29,30,34]. We confirmed the accuracy of the predicted peptide folding with the cristal predicted SWISSMODEL server (https://swissmodel.expasy.org/repository/uniprot/A0A0B8RGN3) and localized the GAPDH-L1 peptide (dark blue in Figure 1, panel (b) and enlarged image) in the homotetramer GAPDH protein of *Listeria monocytogenes* (A0A0B8RGN3_LISM). MHC predictions using the IEDB Consensus tool [36,37,38,39,40] suggested the putative binding epitopes to MHC-I (blue arrows in left images in Figure 1, panel (a) or MHC-II molecules (grey arrows) on the sequences of synthesized GAPDH peptides. The predicted binding epitopes to K^b^ MHC-I molecules were residues 5–13 for LM, MM and SP, and, to D^b^ MHC-I molecules were residues 4–13 (blue arrows, Figure 1, panel (a). The predicted binding epitopes to IA^b^ MHC-II molecules are residues 4–15 and 7–21 for LM, MM and SP (grey arrows) and also residues 8–22 for LM. The predicted MHC-I and MHC-II binding epitopes of GAPDH peptides predicted significant immune responses, while alignments and phylogenetic trees revealed epitope conservation (Supplementary Figure S2).

**Figure 2:**
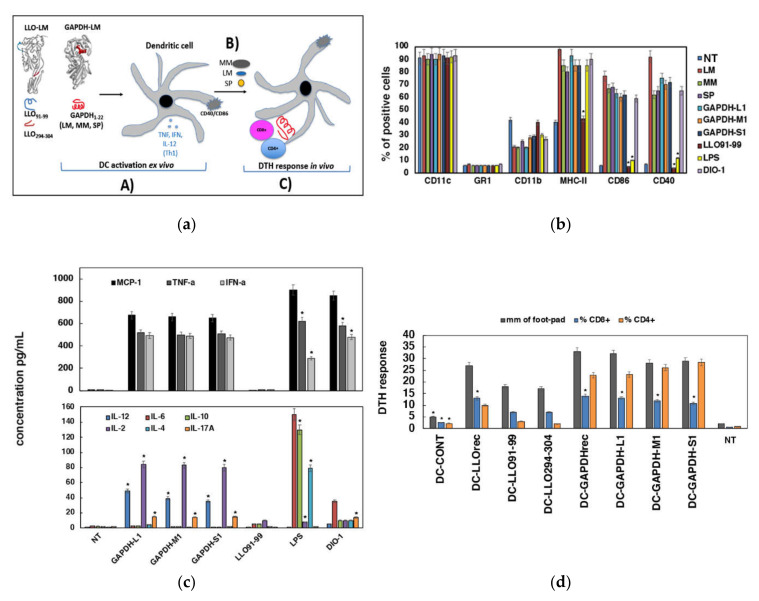
GAPDH-L1, GAPDH-M1 and GAPDH-S1 peptides elicited similar immune responses in DC vaccine vectors. (**a**), Scheme of the approach we have used to examine the efficiency of DC vaccines. First, DC were loaded ex vivo with the antigens to explore DC activation, percentages of CD40^+^ and CD86^+^ positive cells and levels of Th1 cytokines released to supernatants (strategy A). Second, mice were challenged i.v. with the bacteria, LM, MM or SP (strategy B). Third, mice were inoculated with DC vaccines into the left hind footpads, DTH responses were measured and popliteal lymph nodes isolated to analyze T cell populations (strategy C). (**b**)**,** Flow cytometry analysis of DC surface markers after incubation with GAPDH-L1, GAPDH-M1 and GAPDH-S1 peptides. Results indicate the percentages of CD11c^+^, MHC-II^+^, CD40^+^ or CD86^+^ positive cells. Results are the mean of three different experiments ± SD. Student *t*-test was applied for statistics. * *p* < 0.05. (**c**), Cytokine levels released to the supernatants of DC as in (b). Cytokine are measured with a multiparametric CBA kit (BD Biosciences). Results are expressed as pg/mL of each cytokine ± SD of triplicate samples. ANOVA test was applied. * *p* ≤ 0.05. (**d**), C57BL/6 mice were immunized i.v. with 5 × 10^3^ CFU/mice and then left hind footpads were inoculated with 1 × 10^6^ DC vaccines, DC-LLO_rec_, DC-GAPDH_rec_, DC-GAPDH-L1, DC-GAPDH-M1, DC-GAPDH-S1, DC-LLO_91–99_, DC-LLO_294-304_, empty DC or saline (NT) in the presence of 25 µg/mL of DIO-1, while right hind footpads are not inoculated to serve as controls. Footpad swelling was measured with a caliper (grey bars) and expressed as the differences in mm between left and right hind footpads. Results are the mean ± SD of three different experiments. Student *t*-test was applied for statistics. * *p* < 0.05 compared to empty DC samples. Popliteal lymph nodes were isolated from inoculated mice legs and after homogenization, T cells sub-populations were analyzed by flow cytometry. Percentages of CD4^+^ (orange bars) or CD8^+^ T cells (blue bars) are shown. Results are expressed as the percentages of positive cells ± SD of three different experiments. ANOVA test was applied. * *p* < 0.05 compared to empty DC samples.

To validate the immunogenic abilities of GAPDH-L1, GAPDH-M1 and GAPDH-S1 peptides in DC vaccine platforms, we followed the scheme presented in panel (a) of Figure 2 that evaluated two DC functions relevant for eliciting immune responses: (i) DC activation examined in vitro as the adjuvant versus cytotoxic capacities of the peptides, and, by the production of Th1/Th2 cytokines (strategy A in panel (a) of Figure 2) and (ii) DC immunogenic capacities examined by the extend in vivo delayed-type hypersensitivity (DTH) responses (strategy C in panel (a) of Figure 2).

The analysis of cell surface markers of activation such as MHC-II, CD40 or CD80 by flow cytometry, and the levels of Th1/Th2/Th17 cytokines released to DC culture supernatants are two measurements related to DC activation (strategy A in panel (a) of Figure 2). We also included in the assays two adjuvants as positive controls of DC activation, LPS (10 ng/mL) and DIO-1. As it is shown in Figure 2 (panel (b)), incubation of DC with GAPDH-L1, GAPDH-M1 or GAPDH-S1 peptides for 16 h (37 °C) induced high percentages of CD40^+^ or CD80^+^ positive cells, comparable to DC infection with the pathogens or the action of the Th1 adjuvant DIO-1. LPS only induced an intermediate DC activation with high percentages of MHC-II+ cells but low percentages of CD40^+^ and CD80^+^ positive cells. No other peptides induced DC activation, as the listeriosis peptide LLO_91–99_ that presented similar percentages of these cell surface makers as untreated DC (NI). These results indicated that peptides GAPDH-L1, GAPDH-M1 and GAPDH-S1 activated DC in a similar pattern than DIO-1 adjuvant. Next, we evaluated the production of cytokines and confirmed de action of GAPDH-L1, GAPDH-M1 or GAPDH-S1 peptides to induce a classical Th1 cytokine pattern, with high levels of MCP-1, TNF-α, IFN-γ, IL-2, IL-17A and IL-12 (panel (c) of Figure 2). Incubation with LPS induced Th1 but also Th2 cytokines, IL-6, IL-4 and IL-10. We concluded that GAPDH-L1, GAPDH-M1 and GAPDH-S1 peptides behaved as a classical Th1 adjuvant, inducing high percentages of CD40 and CD80 positive DC and promoting the production of high levels of Th1 cytokines. To complete our studies of DC activation, we confirmed that none of the GAPDH peptides, GAPDH-L1, GAPDH-M1, GAPDH-S1, induced cell toxicities or apoptosis on DC (Supplementary Figure S3), including several negative controls such as other GAPDH peptides, GAPDH-L2 (Listeria N-terminal 23–45 amino acids) or GAPDH-Lc (Listeria C-terminal 315–337 amino acids), and other LM peptides as LLO_91–99_. We also included a positive control, recombinant LLO, that induced significant 5% cell toxicity and 12% of DC apoptosis as expected (Supplementary Table S2) [23]. Finally, since DC activation might be induced by endotoxin present in the preparations of the reagents, we confirmed that induction of DC activation by GAPDH-L1, GAPDH-M1 or GAPDH-S1 peptides was not explained by the endotoxin presence in the peptide preparations since they showed less than 0.1. EU/mL using the Genescript ToxiSensor^TM^ chromogenic Limulus amebocyte lysate kit (Cat. No. L0035C).

Next, we explored the immunogenic abilities elicited by DC loaded with GAPDH-L1, GAPDH-M1 or GAPDH-S1 peptides examining the induction in vivo of DTH responses. DC loaded with different GAPDH or LLO peptides or recombinant proteins LLO_rec_ or GAPDH_rec_ as positive controls were inoculated into the left hind footpads of mice, pre-challenged with LM, MM or SP, respectively. DTH responses are measured 48 hours later by the footpad swelling examined with a caliper, as a valid reported method to detect induction of cellular immune responses (strategy C in panel (a) of Figure 2), similarly T cell proliferation was also observed using [^3^H]-thymidine incorporation into T cells after in vitro stimulation with different concentrations of peptides (Supplementary Figure S4) [19]. To explore in detail the immune responses, we isolated popliteal lymph nodes and examined the percentages of CD8^+^ or CD4^+^ T cells by flow cytometry. Our results indicated that the highest DTH responses corresponded to DC vaccines loaded with recombinant protein GAPDH_rec_ or GAPDH peptides (GAPDH-L1, GAPDH-M1 or GAPDH-S1) (grey bars in panel (d) of Figure 2). Interestingly, these high DTH responses correlated with high percentages of CD8^+^ and CD4^+^ T cells in the lymph nodes (blue and orange bars in panel (d) of Figure 2). Recombinant LLO protein, LLO_rec_ or LLO peptides, LLO_91–99_ or LLO_294–304_, induced lower DTH responses and percentages of CD8^+^ and CD4^+^ T cells, especially the LLO peptides. In brief, GAPDH presented higher immunogenic abilities than LLO and DC loaded with GAPDH-L1, GAPDH-M1 or GAPDH-S1 peptides induced very high immunogenic and adjuvant abilities in DC vaccines, comparable to the whole protein, GAPDH_rec_, predicting good efficiency as vaccine epitopes.

### 3.3. Validation of DC Loaded with GAPHD-L1, GAPDH-M1 or GAPDH-S1 Peptides as Cross-Reactive Vaccines

**Figure 3:**
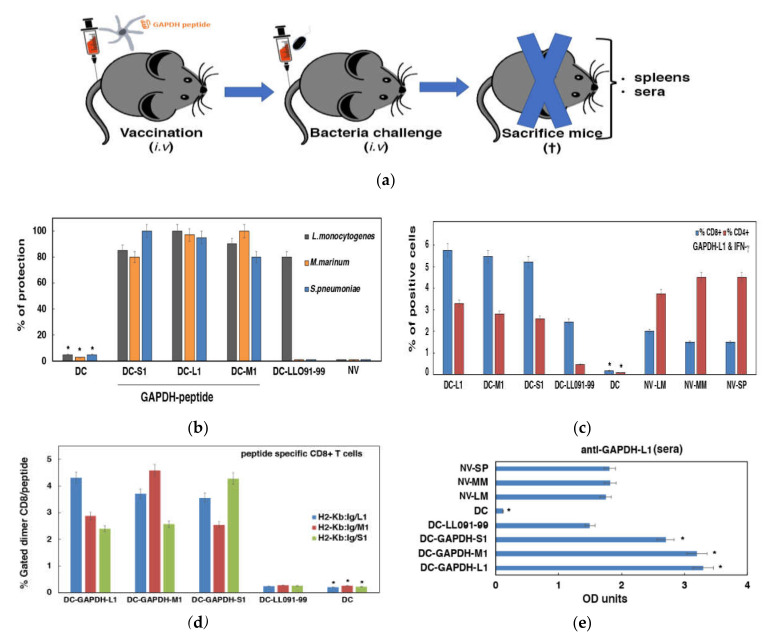
Validation of DC loaded with GAPDH peptides as cross-reactive vaccines for LM, MM or SP infections. (**a**) Vaccination protocol of C57BL/6 mice with a single dose of DC vaccines. Seven days later, each group of vaccinated mice were divided into 3 sets and challenged i.v. with 10^4^ CFU/mice of hypervirulent strains of LM, MM or SP. Next, after 14 days mice were bled, sacrificed and spleens collected. (**b**) Vaccination results expressed percentages of protection as the mean ± SD of triplicates. Percentages are calculated as the number of CFU/mL counted in spleen homogenates of NV mice (saline) divided by CFU/mL of each set of vaccinated mice. Results are expressed as the mean ± SD of triplicates. Student *t*-test was applied for statistics. * *p* < 0.05 compared to DC samples. (**c**) Intracellular cytokines of CD4 and CD8 positive cells specific for GAPDH-L1. Spleen homogenates of DC vaccinated mice or NV were analyzed by flow cytometry and the percentages of CD8^+^ or CD4^+^ T cells are shown. Percentages of CD4^+^ (red) and CD8^+^ (blue) positive cells are expressed as the mean ± SD of triplicates. ANOVA test was applied. * *p* < 0.05 compared to DC samples. (**d**) Frequencies of GAPDH-L1, GAPDH-M1 or GAPDH-S1-specific CD8^+^ T cells induced by DC vaccines are shown. Splenocytes of vaccinated or NV mice were incubated with recombinant dimeric H-2Kb: Ig fusion protein loaded with each peptide. The staining cocktail contained dimeric fusion protein loaded with peptides, CD8 and IFN-γ antibodies. CD8^+^ were gated for anti-IFN-γ staining (%gated dimer-CD8) to calculate the frequencies of CD8^+^-peptide restricted cells and IFN-γ producers. Results are the mean ± SD of triplicates. ANOVA test was applied. * *p* < 0.05 compared to DC samples. (**e**) Sera from vaccinated or NV mice were thawed to check a peptide-ELISA. Reactions were developed with goat anti-mouse IgG and absorbances (OD) analyzed at 450 nm. Results are expressed as the mean ± SD of triplicates. Student *t*-test was applied for statistics. * *p* < 0.05 compared to DC samples.

To confirm our hypothesis that DC loaded with GAPDH-L1, GAPDH-M1 and GAPDH-S1 peptides were efficient vaccines, we used them in mice models of LM, MM or SP infections. We argued that infections with the hypervirulent clinical isolates, HUMV-LM01, HUMV-MM01 or HUMV-SP01, are useful tools to validate DC vaccines efficiency in the most severe conditions. The vaccination scheme is shown in panel (a) of Figure 3.

Mice were vaccinated i.v. with one dose of 10^6^ DC loaded with GAPDH-L1, GAPDH-M1 or GAPDH-S1 (50 µg/mL) or remained non-vaccinated (NV-DC or saline). Seven days later, groups of vaccinated mice (*n* = 15) with DC-GAPDH-L1, DC-GAPDH-M1 or DC-GAPDH-S1 were divided into three sets and each set (*n* = 5) challenged i.v. with a different pathogen (10^4^ CFU/mice of LM, MM or SP). Fourteen days after the bacterial challenge, mice were bled to collect sera and sacrificed to collect spleens. CFU were examined in spleens after homogenization and counting of viable bacteria CFU in BHI agar plates to calculate percentages of protection. Mice vaccinated with DC-GAPDH-L1, DC-GAPDH-M1 or DC-GAPDH-S1 and infected with HUMV-LM01, HUMV-MM01 or HUMV-SP01 clinical isolates, presented percentages of protection higher than 82%, indistinctly. However, mice vaccinated with DC-LLO_91–99_ vaccines induced lower 60–80% percentages of protection and only after HUMV-LM01 challenge but no protection after HUMV-MM01 or HUMV-SP01 bacterial challenge. Non-vaccinated mice (NV) or mice vaccinated with empty vaccine vectors (NV-DC) showed no protection at all against HUMV-01, HUMV-MM01 or HUMV-SP01 challenges (panel (b) of Figure 3). These results strongly suggested that DC-GAPDH-L1, DC-GAPDH-M1 or DC-GAPDH-S1 vaccines induced cross-immune reactions able to confer protection.

We next analyzed the GAPDH-L1 epitope specific immune responses elicited by DC-GAPDH-LM1, DC-GAPDH-MM1 or DC-GAPDH-SP1 vaccines examining the percentages of intracellular cytokines GAPDH-L1-specific. We observed very high percentages of GAPDH-L1-specific and IFN-γ producers of CD8^+^ and CD4^+^ T cells in mice vaccinated with DC vaccines loaded with GAPDH-L1, GAPDH-M1 or GAPDH-S1 (panel (c) in Figure 3). As expected, NV or empty DC vaccinated mice did not induce GAPDH-L1-specific and IFN-γ producers CD4^+^ or CD8^+^ T. We concluded that vaccines inducing cross-reactive immunity seem induced similar to CD4^+^ and CD8^+^ T cell responses for a wide range of epitopes, whenever the epitopes share higher than 98% sequence homology. We also explored the frequencies of CD8^+^ T cells specific for each GAPDH peptide using H2-Kb: Ig fusion dimers. Mice vaccinated with DC-GAPDH-L1 vaccines and challenged with HUMV-LM01 presented very high frequencies, 4.5% of GAPDH-L1-specific CD8^+^ T cells and IFN-γ producers (blue bars in panel (d) of Figure 3) and lower but significant frequencies for GAPDH-M1 (2.8% frequencies of GAPDH-M1-specific CD8^+^ T cells) and GAPDH-S1 2.3% frequencies of GAPDH-S1-specific CD8^+^ T cells). Similarly, the group of mice vaccinated with DC-GAPDH-M1 vaccines and challenged with HUMV-MM01 presented very high frequencies, 4.7% of GAPDH-M1-specific CD8^+^ T cells and IFN-γ producers (red bars in panel (d) of Figure 3) and lower but significant frequencies for GAPDH-L1 (3.7% frequencies of GAPDH-L1-specific CD8^+^ T cells) and GAPDH-S1 (2.3% frequencies of GAPDH-S1-specific CD8^+^ T cells). Finally, the group of mice vaccinated with DC-GAPDH-S1 and challenged with HUMV-SP01 also showed very high frequencies, 4.4% of GAPDH-S1-specific CD8^+^ T cells and IFN-γ producers (green bars in panel (d) of Figure 3) and lower but significant frequencies for GAPDH-L1 (3.8% frequencies of GAPDH-L1 specific CD8^+^ T cells) and GAPDH-M1 (2.5% frequencies of GAPDH-S1-specific CD8^+^ T cells). It seems that GAPDH-L1 induced the highest cross-reactive immune vaccines in all DC vaccines prepared, appearing as the epitope conferring wider protection. DC vaccines loaded with LLO_91–99_ showed no significant frequencies of CD8^+^ T cells specific for any GAPDH peptide, indicating the high specificity of the assay. These results strongly suggested that cross-reactive DC vaccines loaded with GAPDH-L1, GAPDH-M1 or GAPDH-S1 peptides provide two types of T cell responses, high-specific CD8^+^ T cells for the vaccinated epitope, but also broader CD8^+^ T cell responses to GAPDH epitopes sharing more than 98% sequence homology.

Humoral cross-reactive immune responses were also examined in vaccinated and non-vaccinated mice, exploring the anti-GAPDH-L1 antibody titers. Mice vaccinated with DC-GAPDH-L1, DC-GAPDH-M1 or DC-GAPDH-M1 vaccines and challenged either with HUMV-LM01, HUMV-MM01 or HUMV-SP01, presented the highest levels of antibodies, OD ≥ 2.5. Vaccination induced at least 2-fold higher anti-GAPDH-L1 titers than NV mice but challenged with HUMV-LM01, HUMV-MM01 or HUMV-SP01 (panel (e) in Figure 3), indicating that immune responses of vaccinated mice were more powerful than immune responses induced upon pathogen infection. These results suggested that levels of anti-GAPDH-L1 antibodies were good biomarkers of the efficiency of cross-reactive vaccines.

Th1-Th2 cytokines were also analyzed in vaccinated and NV mice. Mice vaccinated with DC-GAPDH-L1, DC-GAPDH-M1 or DC-GADPH-S1 produced high levels of Th1 cytokines, TNF-α, IFN-γ, and IL-12, while they induced low levels of Th2 cytokines, IL-6 and IL-10 (Table 2). Therefore, DC-GAPDH-L1, DC-GAPDH-M1 or DC-GAPDH-S1 vaccination changed the classical Th2 cytokine pattern detected in NV mice challenged with HUMV-LM01, HUMV-MM01 or HUMV-SP01 clinical isolates to a Th1 profile.

## 4. Conclusions

DC vaccines loaded with GAPDH, GAPDH-L1, GAPDH-M1 or GAPDH-S1 peptides appear as cross-reactive vaccines able to confer good protection indistinctly against the following bacterial pathogens, *Listeria monocytogenes*, *Mycobacterium marinum* and *Streptococcus pneumoniae*. The efficiency of DC vaccines loaded with GAPDH-L1, GAPDH-M1 or GAPDH-S1 peptides to provide cross-reactive immunity implies the induction of strong and broad spectrum CD8^+^ T cell responses but limited to epitopes sharing 98% sequence homology. Vaccine efficiency also is accompanied by induction of significant GAPDH-L1-specific CD4^+^ T cell responses, and high titers of anti-GAPDH-L1 antibodies. In fact, as biomarkers of vaccine efficiency easy to evaluate are the high titers of anti-GAPDH-L1 peptide antibodies and the Th1 cytokine profiles. Taken together, these results strongly suggest that the design of effective cross-reactive DC vaccines for listeriosis, cutaneous mycobacteria and Streptococcus should include GAPDH epitopes inducing strong and epitope specific CD8^+^ T cells with a broad spectrum but quoted to epitopes sharing 98% sequence homology. Cross-reactive DC vaccines should include also epitopes that induce significant CD4^+^ T cells. In this regard, GAPDH-L1, GAPDH-M1 and GAPDH-S1 epitopes fulfill all these requirements.

GAPDH based cross-reactive vaccines able to protect against *Listeria*, *Mycobacterium* or *Streptococcus* taxonomic groups might be relevant to design vaccines for adults. In fact, severe infections in the elderly and cancer patients are caused by *Listeria monocytogenes*, *Mycobacterium tuberculosis* or *Streptococcus pneumoniae*, together with acute respiratory virus as Influenza or coronavirus, including SARS-COV-2 [41,42]. Therefore, cross-reactive vaccines might help to decrease the number of mortalities caused in the populations at high risk, induce broad-spectrum immune responses and avoid long antibiotic treatments. We expected that these DC-GAPDH peptide cross-reactive vaccines could offer protection against most, if not all, pathogenic bacteria of the taxonomic groups, *Listeria*, *Mycobacterium* and *Streptococcus*. However, further studies would sustain or not this hypothesis.

## 5. Patents

This study is protected by patent number WO200802108A1. https://patents.google.com/patent/WO2008020108A1/fi#patentCitations (accessed on 16 March 2021), entitled to Fundación Marqués de Valdecilla and patent number WO2019243647. https://patentscope.wipo.int/search/es/detail.jsf?docId=WO2019243647 (accessed on 16 March 2021), entitled to Fundación Instituto de Investigación Marqués de Valdecilla.

## Figures and Tables

**Table 1:** Bacteria isolated at HUMV (2014–2018) with GAPDH 90% sequence homology at the N-terminus after a bioinformatic analysis.

Bacteria	^1^ Number of Isolates (2014–2018)	^2^ Number of Isolates (2016)
* Listeria monocytogenes	27	7
*Mycobacterium avium*	8	1
*Mycobacterium chelonae*	6	5
* Mycobacterium marinum	1	1
*Mycobacterium tuberculosis complex*	64	14
*Mycobacterium tuberculosis*	41	1
*Streptococcus agalactiae*	880	248
* Streptococcus pneumoniae	510	106
* Streptococcus pyogenes	176	31

^1^ Bacterial strains isolated at the Microbiology Department of HUMV from 2014 to 2018, whose GAPDH sequence homologies were higher than 90%. ^2^ Bacterial strains used to calculate annual incidences. * Asterisks correspond to those bacterial strains selected for further analyses. All clinical isolates were isolated from patients older than 50 years of age.

**Table 2:** Specific immune responses elicited after vaccination of mice with DC-GAPDH peptides and challenge with LM (HUMV-LM01), MM (HUMV-MM01) or SP (HUMV-SP01).

^1^ Mice Vaccination	IFN-γ	^2^ CYTOKINESIL-6	IL-10	IL-12
HUMV-LM01 (NV)	4 ± 0.1	20 ± 0.2	16 ± 0.1	2 ± 0.1
DC-GAPDH-L1/LM01	124 ± 0.1	3.4 ± 0.1	1.3 ± 0.1	32 ± 0.2
DC-GAPDH-M1/LM01	112 ± 0.2	7.2 ± 0.2	2.3 ± 0.2	22 ± 0.1
DC-GAPDH-S1/LM01	110 ± 0.3	6.9 ± 0.1	3 ± 0.1	20 ± 0.2
HUMV-MM01 (NV)	4 ± 0.1	10 ± 0.3	8 ± 0.1	1.3 ± 0.1
DC-GAPDH-L1/MM01	106 ± 0.2	3.1 ± 0.1	1.5 ± 0.1	30 ± 0.1
DC-GAPDH-M1/MM01	101 ± 01	2.4 ± 0.1	2 ± 0.2	28 ± 0.2
DC-GAPDH-S1/MM01	102 ± 0.1	3 ± 0.1	2.2 ± 0-1	23 ± 0.2
HUMV-SP01 (NV)	3 ± 0.4	12 ± 0.1	9 ± 0.1	1.2 ± 0.1
DC-GAPDH-L1/SP01	115 ± 0.2	2 ± 0.1	1.3 ± 0.1	29 ± 0.1
DC-GAPDH-M1/SP01	110 ± 0.1	2.5 ± 0.1	2.3 ± 0.1	28 ± 0.1
DC-GAPDH-S1/SP01	120 ± 0.2	2 ± 0.1	1.5 ± 0.1	29 ± 0.1
CONTROL-NI	1 ± 0.1	2 ± 0.1	1.3 ± 0.1	1 ± 0.1

^1^ Female C57BL/6 mice were i.v. vaccinated or not (NV) with DC loaded with different GAPDH peptides (*Listeria monocytogenes* GAPDH-L1, named L1; *Mycobacterium marinum* GAPDH-M1, named M1; *Streptococcus pneumoniae* GAPDH-S1, named S1) and 7 days later all animals were challenged with 5 × 10^3^ CFU bacteria from clinical isolates HUMV-LM01, HUMV-MM01 or HUMV-SP01. Fourteen days later, mice were bled, sacrificed and spleens collected. ^2^ Spleens of mice vaccinated or not were homogenized and cultured cells were used to measure intracellular IFN-γ after GAPDH-L1 peptide stimulation in the presence of brefeldin A (*procedure described in Material and Methods Section 2.11*). The percentages of CD4^+^ or CD8^+^ expressing IFN-γ were determined according with the manufacturer’s recommendations. ANOVA test was applied for statistical analysis. *P* ≤ 0.05.

## Data Availability

No new data were created or analyzed in this study. Data sharing is not applicable to this article.

## Supplementary Materials

The following are available online at https://www.mdpi.com/2076-393X/9/3/269/s1, Figure S1: GAPDH of Listeria, Mycobacterium and Streptococcus are detected in DC and shared enzymatic activity and protein and immunogenic domains; Figure S2: GAPDH of *Listeria*, *Mycobacterium* and *Streptococcus* are detected in DC and shared enzymatic activity and protein and immunogenic domains; Figure S3: Quality controls of DC vaccines; Figure S4: Immunogenicity of DC vaccines loaded with GAPDH peptides; Table S1: Virulence in mice of clinical isolates from the taxonomic groups *Listeria*, *Mycobacterium* and *Streptococcus*.

## References

[1] Córdoba E.V., Pion M., Rasines B., Filippini D., Komber H., Ionov M., Bryszewska M., Appelhans D., Muñoz-Fernández M.A. (2013). Glycodendrimers as new tools in the search for effective anti-HIV DC based immunotherapies. Nanomedicine.

[2] Cohen N., Margalit R., Pevsner-Fischer M., Yona S., Jung S., Eisenbach L., Cohen I.R. (2012). Mouse dendritic cells pulsed with capsular polysaccharide induce resistance to lethal pneumococcal challenge: Roles of T cells and B cells. PLoS ONE.

[3] Rey-Ladino J., Ross A.G.P., Cripps A.W. (2014). Immunity, Immunopathology, and human vaccine development against sexually transmitted Chlamydia trachomatis. Hum. Vaccin. Immunother..

[4] Kawasaki N., Rillahan C.D., Cheng T.Y., Van Rhijn I., Macauley M.S., Moody D.B., Paulson J.C. (2014). Targeted delivery of mycobacterial antigens to human dendritic cells via Siglec-7 induces robust T cell activation. J. Immunol..

[5] Fromen C.A., Robbins G.R., Shen T.W., Kai M.P., Ting J.P., DeSimone J.M. (2015). Controlled analysis of nanoparticle charge on mucosal and systemic antibody responses following pulmonary immunization. Proc. Natl. Acad. Sci. USA.

[6] Kono M., Nakamura Y., Suda T., Uchijima M., Tsujimura K., Nagata T., Giermasz A.S., Kalinski P., Nakamura H., Chida K. (2012). Enhancement of protective immunity against intracellular bacteria using type-1 polarized dendritic cell (DC) vaccine. Vaccine.

[7] Calderon-Gonzalez R., Frande-Cabanes EBronchalo-Vicente L., Lecea-Cuello M.J., Bosch-Martinez A., Fanarraga M.L., Yañez-Diaz S., Carrasco-Marin E., Alvarez-Dominguez C. (2014). Cellular vaccines in listeriosis: Role of the Listeria antigen GAPDH. Front. Cell. Infect. Microbiol..

[8] Martín-Moreno A., Jiménez Blanco J.L., Mosher J., Swanson D.R., García Fernández J.M., Sharma A., Ceña V., Muñoz-Fernández M.A. (2020). Nanoparticle-Delivered HIV Peptides to Dendritic Cells a Promising Approach to Generate a Therapeutic Vaccine. Pharmaceutics.

[9] Mekonnen Z.A., Masavuli M.G., Yu W., Gummow J., Whelan D.M., Al-Delfi Z., Torresi J., Gowans E.J., Grubor-Bauk B. (2020). Enhanced T Cell Responses Induced by a Necrotic Dendritic Cell Vaccine, Expressing HCV NS3. Front. Microbiol..

[10] Han J., Sun J., Zhang G., Chen H. (2020). DCs-based therapies: Potential strategies in severe SARS-CoV-2 infection. Int. J. Med. Sci..

[11] Pagliano P., Arslan F., Ascione T. (2017). Epidemiology and treatment of the commonest form of listeriosis: Meningitis and bacteraemia. Infez. Med..

[12] Marais B.J., Heemskerk A.D., Marais S.S., van Crevel R., Rohlwink U., Caws M., Meintjes G., Misra U.K., Mai N.T., Ruslami R. (2017). Standarized methods for enhanced quality and comparability of tuberculous meningitis studies. Clin. Infect. Dis..

[13] Herrador Z., Gherasim A., Lopez-Velez E., Benito A. (2019). Listeriosis in Spain based on hospitalization records 1997 to 2015: Need for greater awareness. Eurosurveillance.

[14] McGill F., Heyderman R.S., Panagiotou S., Tunkel A.R., Solomon T. (2016). Acute bacterial meningitis in adults. Lancet.

[15] Gonzalez-Santiago T.M., Drage L.A. (2015). Nontuberculous Mycobacteria. Skin and soft tissue infections. Dermatol. Clin..

[16] Godshall C.E., Suh G., Lorber B. (2013). Cutaneous listeriosis. J. Clin. Microbiol..

[17] Johansson L., Thulin P., Low D.E., Norrby-Teglund A. (2010). Getting under the skin: The immunopathogenesis of Streptococcus pyogenes deep tissue infections. Clin. Infect. Dis..

[18] Weinberger B. (2018). Vaccines for the elderly: Current use and future challenges. Immun. Ageing.

[19] Alvarez-Dominguez C., Madrazo-Toca F., Fernandez-Prieto L., Vandeckerhove J., Pareja E., Tobes R., Gomez-Lopez M.T., Del Cerro-Vadillo E., Fresno-Escudero M., Leyva-Cobián F. (2008). Characterization of a Listeria monocytogenes protein interfering with Rab5a. Traffic.

[20] Calderon-Gonzalez R., Tobes R., Pareja E., Frande-Cabanes E., Alaez-Alvarez L., Petrovsky N., Alvarez-Dominguez C. (2015). Identification and characterisation of T-cell epitopes for incorporation into dendritic cell-delivered *Listeria* vaccines. J. Immunol. Methods.

[21] Alvarez-Dominguez C., Carrasco-Marin E. Peptides Which Are Immunogenic in Relation to the Genuses Listeria and Mycobacterium, Antibodies and Uses of these. Patent Number.

[22] Alvarez-Dominguez C., Calderon-Gonzalez R., Teran-Navarro H., Salcines-Cuevas D., Frande-Cabanes E., Garcia I., Marradi M., Penades-Ullate S. Multivalent Vaccine for the Treatment and Prevention of Tuberculosis, Listeriosis and Pneumonia. Patent Number.

[23] Alvarez-Dominguez C., Salcines-Cuevas D., Teran-Navarro H., Calderon-Gonzlez R., Tobes R., Garcia I., Grijalvo S., Paradela A., Seoane A., Sangari F.J. (2020). Epitopes for multivalent vaccines against Listeria, Mycobacterium and Streptococcus spp: A novel role for glyceraldehyde-3-phosphate dehydrogenase. Front. Cell. Infect. Microbiol..

[24] Alvarez-Dominguez C., Stahl P.D. (1999). Increased expression of Rab5a correlates directly with accelerated maturation of Listeria monocytogenes phagosomes. J. Biol. Chem..

[25] Barbieri M.A., Sha Q., Bette-Bobillo P., Stahl P.D., Vidal M. (2001). ADP-ribosylation of Rab5 by ExoS of Pseudomonas aeruginosa affects endocytosis. Infect. Immun..

[26] Ovejero-Guisasola J.I., Fresno-Escudero M. Lipopolysaccharide of Ochrobactrum Intermedium and Their Use as Immunostimulant of Mamalians. Patent Number.

[27] Calderon-Gonzalez R., Frande-Cabanes E., Teran-Navarro H., Marimon J.M., Freire J., Salcines-Cuevas D., Fariñas M.C., Gonzalez-Rico C., Marradi M., Garcia I. (2017). GNP-GAPDH_1-22_ nanovaccines prevent neonatal listeriosis by blocking microglia apoptosis and bacterial dissemination. Oncotarget.

[28] Calderon-Gonzalez R., Teran-Navarro H., Frande-Cabanes E., Fernandez-Ferrandez E., Freire J., Penades S., Marradi M., Garcia I., Gomez-Roman J., Yañez-Diaz S. (2016). Pregnancy vaccination with gold glyco-nanoparticles carrying Listeria monocytogenes peptides protects against listeriosis and brain- and cutaneous-associated morbidities. Nanomaterials.

[29] Blum M., Chang H.Y., Chuguransky S., Grego T., Kandasaamy S., Mitchell A., Nuka G., Paysan-Lafosse T., Qureshi M., Raj S. (2021). The InterPro protein families and domains database: 20 years on. Nucleic Acids Res..

[30] Peters B., Sette A. (2005). Generating quantitative models describing the sequence specificity of biological processes with the stabilized matrix method. BMC Bioinform..

[31] Waterhouse A., Bertoni M., Bienert S., Studer G., Tauriello G., Gumienny R., Heer F.T., de Beer T.A.P., Rempfer C., Bordoli L. (2018). SWISS-MODEL: Homology modelling of protein structures and complexes. Nucleic Acids Res..

[32] Guex N., Peitsch M.C., Schwede T. (2009). Automated comparative protein structure modeling with SWISS-MODEL and Swiss-PdbViewer: A historical perspective. Electrophoresis.

[33] Bienert S., Waterhouse A., de Beer T.A.P., Tauriello G., Studer G., Bordoli L., Schwede T. (2017). The SWISS-MODEL Repository—New features and functionality. Nucleic Acids Res..

[34] Lamiable A., Thévenet P., Rey J., Vavrusa M., Derreumaux P., Tufféry P. (2016). PEP-FOLD3: Faster de novo structure prediction for linear peptides in solution and in complex. Nucleic Acids Res..

[35] Kim Y., Ponomarenko J., Zhu Z., Tamang D., Wang P., Greenbaum J., Lundegaard C., Sette A., Lund O., Bourne P.E. (2012). Immune epitope database analysis resource. Nucleic Acids Res..

[36] Sidney J., Assarsson E., Moore C., Ngo S., Pinilla C., Sette A., Peters B. (2008). Quantitative peptide binding motifs for 19 human and mouse MHC class I molecules derived using positional scanning combinatorial peptide libraries. Immunome Res..

[37] Nielsen M., Lundegaard C., Worning P., Lauemøller S.L., Lamberth K., Buus S., Brunak S., Lund O. (2003). Reliable prediction of T-cell epitopes using neural networks with novel sequence representations. Protein Sci..

[38] Lundegaard C., Lamberth K., Harndahl M., Buus S., Lund O., Nielsen M. (2008). NetMHC-3.0: Accurate web accesible predictions of Human, Mouse, and Monkey MHC class I affinities for peptides of length 8-11. Nucleic Acids Res..

[39] Andreatta M., Nielsen M. (2016). Gapped sequence alignment using artificial neural networks: Application to the MHC class I system. Bioinformatics.

[40] Vita R., Mahajan S., Overton J.A., Dhanda S.K., Martini S., Cantrell J.R., Wheeler D.K., Sette A., Peters B. (2018). The Immune Epitope Database (IEDB): 2018 update. Nucleic Acids Res..

[41] Solana R., Pawelec G., Tarazona R. (2006). Aging and innate immunity. Immunity.

[42] Sanchez-Ramon S., Conejero L., Netea M.G., Sancho D., Palomares O., Subiza J.L. (2018). Trained immunity based-vaccines: A new paradigm for the development of broad-spectrum anti-infectious formulations. Front. Immunol..

