# Supplementary material for: Glyceraldehyde-3-phosphate Dehydrogenase Common Peptides of Listeria monocytogenes, Mycobacterium marinum and Streptococcus pneumoniae as Universal Vaccines
Source: Vaccines (Basel). 2021 Mar 17;9(3):269. doi: 10.3390/vaccines9030269 (PMC8002646; doi:10.3390/vaccines9030269)
Supplement: Supplementary file 1 [file vaccines-09-00269-s001.pdf]

vaccines

MDPI

SupplementaryMaterials:

Glyceraldehyde-3-phosphate dehydrogenase common peptides of *Listeria monocytogenes, Mycobacterium marinum* and *Streptococcus pneumoniae* as universal vaccines

Virulence study in mice of the bacterial strains isolated in 2016 corresponding to the taxonomic groups of *Listeria, Mycobacterium* and *Streptococcus.* Virulence was explored in C57BL/6 mice after intravenously (*i.v*) infection with 10^4 CFU/mice (*n* = 5). 14 days later, mice are bled, sacrificed and spleens collected to count viable bacteria (CFU/mL) using agar plates. Non-virulent strains of each bacterial genera were included in the assay as basal controls, a listeriolysin deficient mutant of LM (LM-ΔLLO), a non-pathogenic strain of mycobacteria (*Mycobacterium smegmatis*) and a vaccine strain of *Streptococcus pneumoniae* (ATCC 49619-19F). Hypervirulent strains showed at least 100-fold higher CFU numbers in spleens than non-pathogenic strains and high titers of anti-GAPDH-L1 antibodies in sera with OD > 2.0 (*Table S1*).

Table 1. Virulence in mice of clinical isolates from taxonomic groups of Listeria, Mycobacterium and Streptococcus.

[a]Isolate code Period 2014-18	Anti-GAPH antibodies[c]	[b]Analysis in mice Virulence (CFU/mL)[d]	CD4% vs. control[e]	CD8% vs. control
*HUMV-LM01	2.85 ± 0.1	$2.9 \times 10^5 \pm 10$	12 ± 0.2	17 ± 0.3
HUMV-LM02	2.31 ± 0.2	$2.7 \times 10^5 \pm 11$	14 ± 0.3	17 ± 0.2
HUMV-LM03	2.01 ± 0.1	$2.5 \times 10^5 \pm 12$	13 ± 0.2	19 ± 0.3
HUMV-LM04	1.90 ± 0.1	$2.2. \times 10^5 \pm 12$	12 ± 0.3	17 ± 0.2
HUMV-LM05	1.92 ± 0.1	$2.2 \times 10^5 \pm 10$	15 ± 0.2	19 ± 0.3
HUMV-LM06	1.94 ± 0.2	$2.2 \times 10^5 \pm 10$	14 ± 0.3	17 ± 0.3
HUMV-LM07	1.98 ± 0.2	$2.4 \times 10^5 \pm 11$	14 ± 0.3	17 ± 0.4
LM^{WT} (10403S basal control)	*0.89 ± 0.2*	*$2.5 \times 10^2 \pm 12$*	*9 ± 0.1*	*10 ± 0.2*
LM-ΔLLO (non-virulent control)	*0.42 ± 0.1*	*$4.2 \times 10^0 \pm 10$*	*8 ± 0.1*	*1 ± 0.1*
*HUMV-MTB01	2.2 ± 0.1	$3.9 \times 10^4 \pm 10$	18 ± 0.2	14 ± 0.3
HUMV-MA01	1.82 ± 0.2	$3.1 \times 10^4 \pm 12$	17 ± 0.2	15 ± 0.4
*HUMV-MM01	2.15 ± 0.1	$3.8 \times 10^4 \pm 11$	18 ± 0.2	14 ± 0.3
HUMV-MC01	1.75 ± 0.2	$2.0 \times 10^4 \pm 10$	19 ± 0.2	13 ± 0.2
HUMV-MC02	1.65 ± 0.2	$2.0 \times 10^4 \pm 11$	17 ± 0.2	14 ± 0.3
HUMV-MC03	1.61 ± 0.1	$2.2 \times 10^4 \pm 12$	18 ± 0.2	13 ± 0.2
HUMV-MC04	1.64 ± 0.2	$2.0 \times 10^4 \pm 11$	19 ± 0.2	15 ± 0.3
HUMV-MC05	1.63 ± 0.1	$2.1 \times 10^4 \pm 11$	18 ± 0.2	14 ± 0.2
M.smegmatis (non-virulent control)	*0.7 ± 0.2*	*$1.5 \times 10^2 \pm 8$*	*9 ± 0.1*	*1 ± 0.1*
*HUMV-SP01	2.21 ± 0.1	$4.8 \times 10^5 \pm 13$	12 ± 0.2	13 ± 0.3
HUMV-SP02	1.71 ± 0.1	$3.5 \times 10^5 \pm 16$	13 ± 0.3	15 ± 0.4
HUMV-SP03	1.73 ± 0.1	$3.3 \times 10^5 \pm 15$	14 ± 0.4	14 ± 0.3
HUMV-SA01	2.13 ± 0.1	$3.8 \times 10^5 \pm 13$	13 ± 0.3	16 ± 0.3
HUMV-SA02	2.20 ± 0.1	$3.9 \times 10^5 \pm 12$	14 ± 0.3	16 ± 0.4
HUMV-SA03	2.12 ± 0.2	$3.8 \times 10^5 \pm 11$	13 ± 0.3	16 ± 0.4
HUMV-SPY01	1.85 ± 0.1	$4.1 \times 10^5 \pm 17$	12 ± 0.2	13 ± 0.3
HUMV-SPY02	1.90 ± 0.1	$4.0 \times 10^5 \pm 12$	12 ± 0.2	14 ± 0.4
HUMV-SPY03	1.87 ± 0.2	$4.2 \times 10^5 \pm 12$	12 ± 0.2	13 ± 0.3
S.pneumoniae 49619-19F (non-virulent)	*0.67 ± 0.1*	*$1.2 \times 10^3 \pm 10$*	*9 ± 0.2*	*1 ± 0.1*
CONTROL-NI	0.1 ± 0.2	0.06 ± 0.1	0.3 ± 0.1	0.5 ± 0.1

[a] Clinical isolates in the year 2016 from patients older than 50 years of age and infected with the following strains of *L. monocytogenes* (*HUMV-LM01-HUMV-07, 7 isolates), different mycobacteria (M. *tuberculosis*, HUMV-MTB01; *M. avium*, HUMV-MA01; *M. marinum*, *HUMV-MM01 and *M.*

chelonae, HUMV-MC01-HUMV-05) or different streptococci (*S. pneumoniae*, *HUMV-SP01-HUMV-SP03; *S. agalactiae*, HUMV-SA01-HUMV-SA03 and *S. pyogenes*, HUMV-SPY01-HUMV-SPY03) and selected from the 2014-2018 study (*Table S1*). Non-pathogenic LM-ΔLLO, *M. smegmatis* and *S. pneumoniae* 49619-19F strains were also included in the assay as non-virulent controls. [b] Female C57BL/6 mice (n = 5) were *i.v.* inoculated with 10^4 CFU/mice from clinical isolates detailed in *a*. 14 days later, mice were bled, sacrificed and spleens collected. [c] Sera from mice as in *b* were examined for anti-GAPDH-L1 antibodies by a peptide ELISA. Results are presented as the mean ± SD of OD units in triplicate experiments ($p < 0.05$). [d] Spleens from mice as in *b* were homogenized and plated in blood agar plates. CFU were counted and results expressed as CFU/mL ($p \leq 0.5$). Asterisks correspond to the selected clinical isolates for our further study. 10403S LM strain has a LD50 in C57BL/6 of 10^5 CFU/mice, MM LD50 was 2×10^5 CFU/mice and SP LD50 was 10^4 CFU/mice. To equalize infections, we gave a single dose of 10^4 CFU/mice. [e]Percentages of positive cells compared to controls and analyzed by FACS.

Biochemical analysis of GAPDH in DC cells infected with *Listeria monocytogenes, Mycobacterium marinum or Streptococcus pneumoniae*. Bioinformatic predictions of GAPDH sequences of the taxonomic groups *Listeria, Mycobacterium* and *Streptococcus* revealed that the N-terminal presented higher than 95% sequence homology at the Rossman fold domain, the NAD-binding domain [23]. In fact, this NAD-binding domain contained the reported ADP-ribosylation activity onto Rab5a [19]. To explore biochemically if Mycobacterium and Streptococcus also showed GAPDH proteins able to bind to Rab5a, we infected DC with different bacteria: *Listeria monocytogenes* (LM), *Mycobacterium tuberculosis* (MTB), *Mycobacterium marinum* (MM), *Streptococcus pneumoniae* (SP) and non-pathogenic *Escherichia coli* (Ec) and isolated GAPDH proteins using GST-Rab5a columns. Proteins eluted from Rab5a columns presented a molecular weight reported for GAPDH-LM, 43-46 kDa (*panel a of Fig. S1*) [19, 23] that after sequencing we confirmed GAPDH identity. We also detected another band of 56 kDa molecular weight that did not correspond to any LM, MTM, MM or SP peptide map (panel a in *Fig.e S1*). Protein eluted from Blue-sepharose NAD-affinity columns were also sequenced and confirmed they contained GAPDH as well as another protein, enolase for LM, MTB, MM and SP, while MTB also presented two additional proteins, Hsp70 and EF-60. GAPDH eluted from GSP-Rab5, NAD-affinity columns was also detected by western-blot using anti-GAPDH-L1 antibodies (lower gel in panel b of Figure. S1), as well as in DC infected with LM, MM or SP (panel c of Figure. S1).

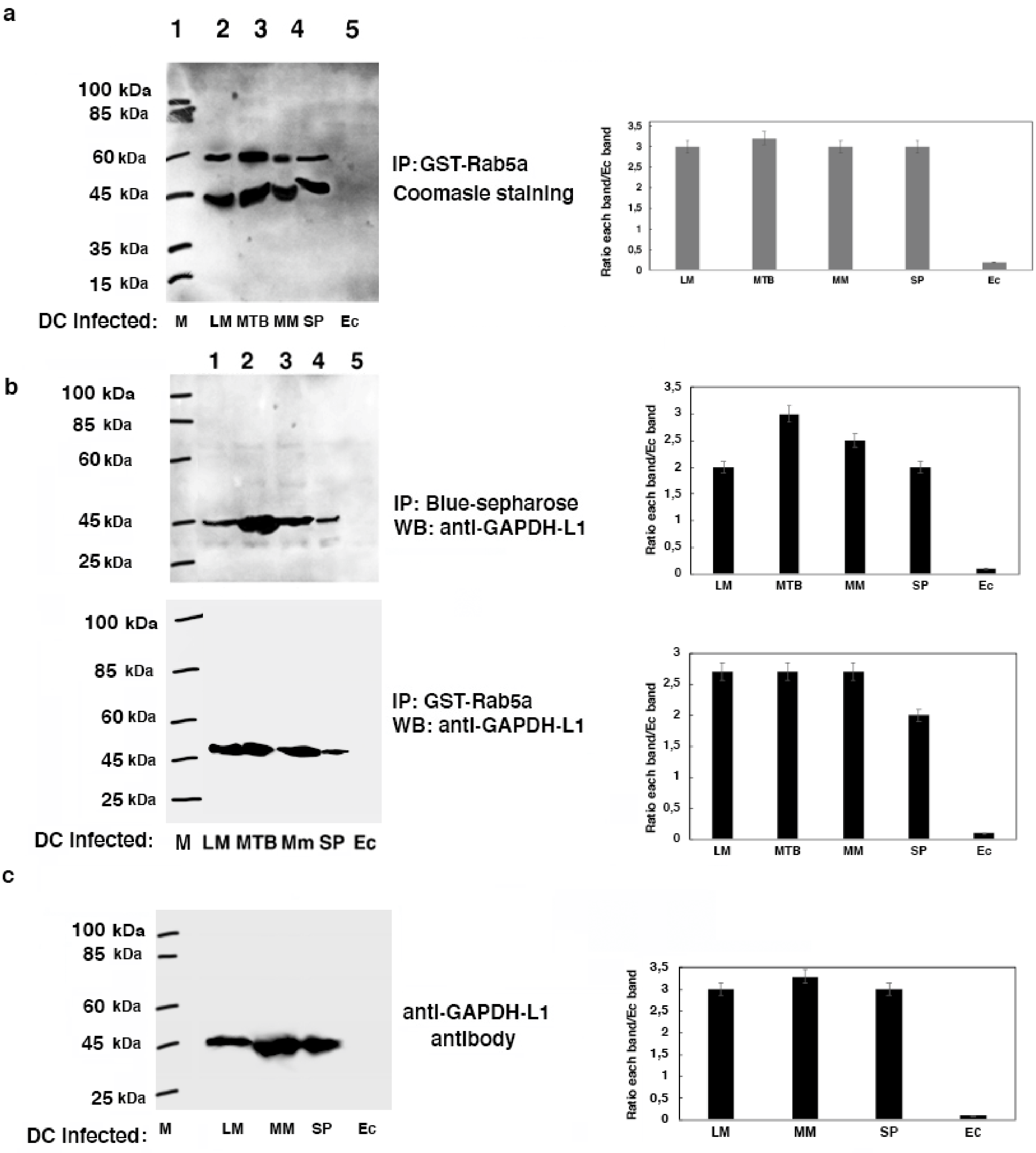

Figure S1. - GAPDH of *Listeria, Mycobacterium* and *Streptococcus* are detected in DC and shared enzymatic activity and protein and immunogenic domains. (**a**) GAPDH isolation and proteomic characterization after DC loading with bacterial extracts of LM, MTB, MM or SP and immunoprecipitation of DC lysates with GST-Rab5a columns. (**b**) DC were infected with different bacterial pathogens: LM, MTB, MM or SP or non-pathogenic bacteria as Ec for 16 hours. A set of lysates of infected DC were immunoprecipitated with Blue-sepharose to isolate NAD-binding proteins and another set immunoprecipitated with GST-Rab5a. Both immunoprecipitations were run on SDS-PAGE gels and western-blot developed with a rabbit anti-GAPDH-L1 antibody that recognized the L1 peptide. (**c**) Bacterial extracts of LM, MM, SP or Ec were run under

SDS-PAGE gels and western-blot developed with rabbit anti-GAPDH-L1 antibody. Plots correspond to intensity ratios of each band compared to Ec band.

We concluded that GAPDH from DC infected with LM, MTB, MM or SP presented same NAD and Rab5a binding domains, ADP-ribosylation enzymatic activities and immunogenic domains. Therefore, they can be considered similar virulent factors of these bacteria.

GAPDH peptide alignments and phylogenetic trees. To explore the epitopes conservation in GAPDH peptides (GAPDH-L1, GAPDH-M1 or GAPDH-S1) we performed GAPDH peptide alignments of different GAPDH sequences from clinical isolates reported in databases. We also performed their phylogenetic trees. The results indicated highly conserved sequences among different clinical isolates as well as epitopes conservation.

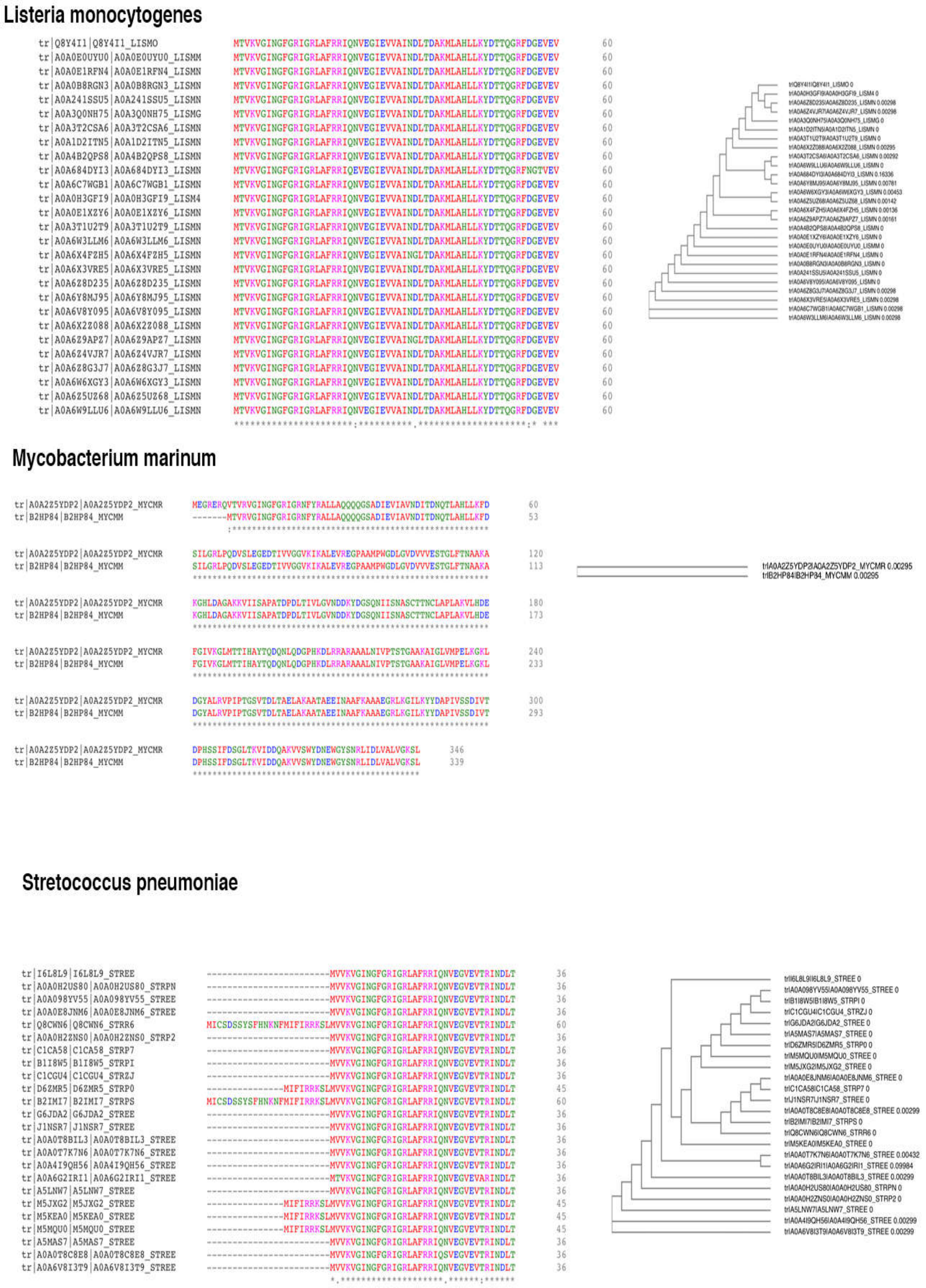

Figure S2. GAPDH peptides alignments and phylogenetic trees. GAPDH sequences reported for different bacterial strains from *Listeria monocytogenes, Mycobacterium marinum* or *Streptococcus pneumoniae* were available at NCBI databases.

Multiple alignment and phylogenetic trees were done using CLUSTAL Omega at EBI server (https://www.ebi.ac.uk/Tools/msa/clustalo/) (accessed on 3 March 2021) [29–34]. Phylogenetic trees were obtained selecting Neighbour-joining algorithm without distance corrections. Explanations about the meaning of the colors in the alignment, the symbols used in the consensus line and about the phylogenetic trees are available at https://www.ebi.ac.uk/seqdb/confluence/display/JDSAT/Bioinformatics+Tools+FAQ (accessed on: 3 March 2021). These results confirmed that GAPDH peptide sequences were highly conserved among different bacterial strains as well as the phylogenetic trees indicated epitopes were highly conserved too.

Cell toxicity and quality controls of prepared DC vaccine vectors. Differentiated DC were loaded with recombinant proteins or peptides to explore toxicities and induction of apoptosis. Recombinant proteins were LLO or GAPDH and peptides GAPDH peptides of *Listeria, Mycobacterium* or *Streptococcus*. Only recombinant LLO induced toxicities and apoptosis.

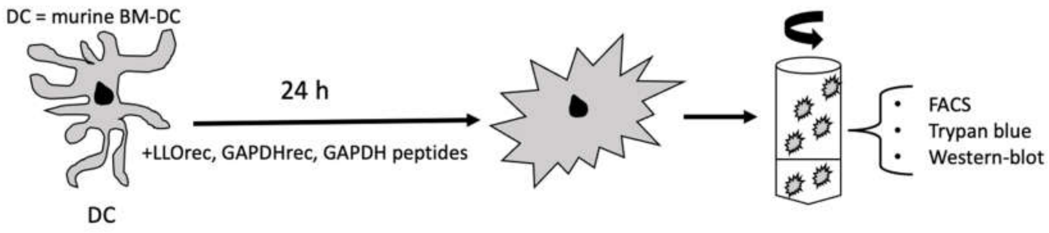

Figure S3. Strategy to explore quality controls of DC loaded with recombinant proteins or peptides. Table present the data of the quality controls.

[1] Samples tested	[2] Cell viability (% Trypan blue+ cells)	[3] Apoptosis (anti-annexin V antibodies)
DC + saline	2.0% ± 0.3	2.0% ± 0.1
DC + LLOrec	1.5% ± 0.6	12% ± 0.9
DC + GAPDHrec	2.9% ± 0.7	2.1% ± 0.2
DC + GAPDH-L1	3.0% ± 0.5	1.8% ± 0.1
DC + GAPDH-L2	2.9% ± 0.7	2.3% ± 0.2
DC + GAPDH-Lc	2.5% ± 0.6	2.4% ± 0.3
DC + GAPDH-M1	2.9% ± 0.7	1.7% ± 0.2
DC + GAPDH-S1	2.45% ± 0.5	1.9% ± 0.2
DC + LLO91-99	2.68% ± 0.4	2.1% ± 0.1

[1] Quality controls of DC vaccine loaded with either recombinant proteins or peptides by two procedure: [2] toxicities explored by Trypan blue experiments that accumulates in nuclei and cytoplasm in non-viable cells and [3] apoptosis, examined by FACS analysis with anti-Ann V antibodies-PE labelled.

Immunogenicity of DC loaded with peptides. DC vaccines were loaded with the different peptides, GAPDH-L1, GAPDH-M1 and GAPDH-S1, as well as with recombinant proteins, LLO or GAPDH. DC vaccines were inoculated into the hint footpads of mice. Poplyteal lymph nodes are recovered, homogenized and in vitro stimulated with different concentrations of the peptides from 0.05 μM to 500 μM and examined general immune responses by a classical proliferation assay of lymphocytes using [^{3}H]-thymidine. Peptides GAPDH-L1, GAPDH-M1 and GAPDH-S1 elicited T cell responses similar to recombinant LLO or GAPDH. We concluded that all peptides elicited T cell responses in the same range than highly immunogenic bacterial proteins, such as LLO or GAPDH.

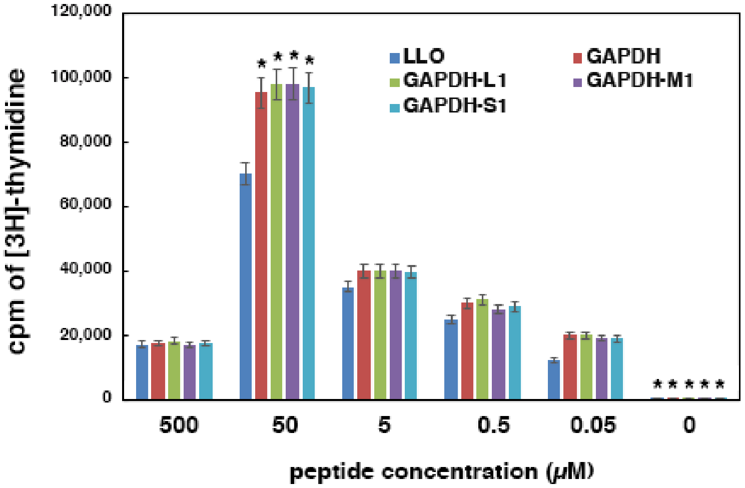

Figure S4. Immunogenicity of DC vaccines loaded with GAPDH peptides. DC loaded with the different GAPDH peptides (GAPDH-L1, GAPDH-M1 or GAPDH-S1) were inoculated into the right hint footpad of mice. Popliteal lymph nodes were collected, homogenated and cultured in vitro in the presence of each corresponding peptide at different concentrations. Plot shows the T cell proliferation after [^{3}H]-thymidine incorporation.
